# Affinity Isolation and Mass Spectrometry Identification of Prostacyclin Synthase (PTGIS) Subinteractome

**DOI:** 10.3390/biology8020049

**Published:** 2019-06-20

**Authors:** Pavel V. Ershov, Yuri V. Mezentsev, Arthur T. Kopylov, Evgeniy O. Yablokov, Andrey V. Svirid, Aliaksandr Ya. Lushchyk, Leonid A. Kaluzhskiy, Andrei A. Gilep, Sergey A. Usanov, Alexey E. Medvedev, Alexis S. Ivanov

**Affiliations:** 1 Department of Proteomic Research and Mass Spectrometry, Institute of Biomedical Chemistry (IBMC), 10 Pogodinskaya str., 119121 Moscow, Russia; yu.mezentsev@gmail.com (Y.V.M.); a.t.kopylov@gmail.com (A.T.K.); evgeyablokov1988@mail.ru (E.O.Y.); la-kaluzhskiy@yandex.ru (L.A.K.); professor57@yandex.ru (A.E.M.); professor-ivanov@yandex.ru (A.S.I.); 2 Laboratory of Molecular Diagnostics and Biotechnology, Institute of Bioorganic Chemistry of the National Academy of Sciences of Belarus, 5, bld. 2 V.F. Kuprevich str., 220141 Minsk, Belarus; svirid.andrei@gmail.com (A.V.S.); lushchik88@gmail.com (A.Y.L.); agilep@yahoo.com (A.A.G.); sergey_usanov@hotmail.com (S.A.U.)

**Keywords:** prostacyclin synthase, protein–protein interactions, surface plasmon resonance, mass spectrometry, size-exclusion chromatography, subinteractome, direct molecular fishing, isatin

## Abstract

Prostacyclin synthase (PTGIS; EC 5.3.99.4) catalyzes isomerization of prostaglandin H_2_ to prostacyclin, a potent vasodilator and inhibitor of platelet aggregation. At present, limited data exist on functional coupling and possible ways of regulating PTGIS due to insufficient information about protein–protein interactions in which this crucial enzyme is involved. The aim of this study is to isolate protein partners for PTGIS from rat tissue lysates. Using CNBr-activated Sepharose 4B with covalently immobilized PTGIS as an affinity sorbent, we confidently identified 58 unique proteins by mass spectrometry (LC-MS/MS). The participation of these proteins in lysate complex formation was characterized by SEC lysate profiling. Several potential members of the PTGIS subinteractome have been validated by surface plasmon resonance (SPR) analysis. SPR revealed that PTGIS interacted with full-length cytochrome P450 2J2 and glutathione S-transferase (GST). In addition, PTGIS was shown to bind synthetic peptides corresponding to sequences of for GSTA1, GSTM1, aldo-keto reductase (AKR1A1), glutaredoxin 3 (GLRX3) and histidine triad nucleotide binding protein 2 (HINT2). Prostacyclin synthase could potentially be involved in functional interactions with identified novel protein partners participating in iron and heme metabolism, oxidative stress, xenobiotic and drugs metabolism, glutathione and prostaglandin metabolism. The possible biological role of the recognized interaction is discussed in the context of PTGIS functioning.

## 1. Introduction

Prostacyclin synthase (PTGIS; EC 5.3.99.4), also known as prostaglandin I_2_ synthase, catalyzes the reaction of prostaglandin H_2_ isomerization to prostacyclin (or prostaglandin I_2_, PGI_2_). PTGIS is characterized by tissue specificity; according to the GeneCards database [https://www.genecards.org], it is mainly determined in tonsil, lung, heart, urinary bladder, uterus, testis. PTGIS co-localizes with cyclooxygenase in the endoplasmic reticulum, plasma membrane and nuclear membrane [1]. 

The functional activity of this clinically relevant enzyme is important for regulation of platelet aggregation and vascular tone, as prostacyclin is a vasodilator and inhibitor of platelet aggregation. An imbalance between prostacyclin and its physiological antagonist thromboxane A_2_ contributes to the development of myocardial infarction, stroke, and atherosclerosis [2,3,4,5]. Certain evidence exists that PTGIS and thromboxane synthase (TBXAS1) may be involved in carcinogenesis [6,7]. 

PGI_2_ is the major metabolite of arachidonic acid in vascular tissue, heart, uterus and gastric mucosa. Similar to thromboxane A_2_, prostacyclin is chemically unstable (half-life of several minutes) and is spontaneously broken down to 6-keto-prostaglandin F1 alpha [8]. We hypothesized that close functional coupling of PTGIS with other proteins involved in prostaglandin metabolism, for example, cyclooxygenase 2 (COX2) [9], as well as the very limited lifetime of most prostaglandin synthase products due to their instability [8,10,11], may indicate the existence of protein partners either directly interacting with PTGIS, or protein complexes forming a protein network required for channeling and the action of short-lived prostaglandins. Such proteins could be attributed to functional enzymes, auxiliary, regulatory and transport proteins and molecular targets for prostaglandin/prostacyclin action. Insufficient information about the real and potential protein–protein interactions of PTGIS in the literature implies a lack of proper understanding of its functional coupling and possible ways of regulation. The following protein partners of PTGIS are mentioned in the interactome databases: caveolin-1 (CAV1), prostaglandin G/H synthase 2 (PTGS2 or COX2) [Funcoup database, http://funcoup.sbc.su.se]; prostaglandin G/H synthase 1 (PTGS1), PTGS2, squalene monooxygenase (SQLE), methyl sterol monooxygenase 1 (MSMO1), cholesterol 25-hydroxylase (CH25H), alkylglycerol monooxygenase (AGMO), lanosterol oxidase (SC5DL), squalene synthase (FDFT1), lanosterol synthase (LSS) [STRING database, score value >0.75, https://string-db.org/]; CAV1, amyloid-beta A4 protein (APP) [BioGRID, https://thebiogrid.org/]; lysine-specific demethylase 5B (KDM5B) [IntAct, https://www.ebi.ac.uk/]; 14-3-3 protein epsilon (Ywhae) [MINT, https://mint.bio.uniroma2.it]. The existence of many protein partners for PTGIS may indicate multiple functions. 

We have previously demonstrated the applicability of different approaches to direct molecular fishing [12,13,14] for isolation and identification of previously unknown and real protein partners for the bait proteins from tissue lysates. These approaches include a combination of paramagnetic nanoparticle technology, affinity chromatography, LC/MS-MS and surface plasmon resonance (SPR) optical biosensors [12,15,16,17]. The present study is aimed at the isolation and identification of new protein partners of prostacyclin synthase using direct molecular fishing combined with size exclusion chromatography (SEC). For this purpose, we used rat tissue lysates and highly purified recombinant protein preparations. 

## 2. Materials and Methods

### 2.1. Recombinant Proteins

#### 2.1.1. Prostacyclin Synthase (PTGIS)

*E. coli* strain BL21(DE3) was transformed with recombinant vector pCW-PTGIS, carrying a sequence of truncated and His-tagged PTGIS or pCW-PTGIS, carrying a sequence of truncated (by the membrane domain) and His-tagged PTGIS with an Avi-tag for in vivo biotinylation. The bacteria were grown overnight in 10 mL LB (with 100 µg/mL ampicillin) at 37 °C. This overnight culture was used to inoculate one liter of fresh TB medium supplemented with 50 µM D-biotin in the case of pCW-PTGIS) and cells were grown at 37 °C with shaking at 180 rpm. Protein expression was induced by adding isopropyl-beta D1-thiogalactopyranoside (IPTG) to a final concentration of 0.5 mM. The culture temperature was reduced to 26 °C following induction and cells were harvested by centrifugation at 3000× *g* for 10 min. The protein was purified on Ni–NTA resin following size-exclusion chromatography (SEC) on HiLoad 26/60 Superdex 200 pg column. Highly purified PTGIS preparation (>95% according to SDS-PAGE) was stored at −75 °C for further use in buffer containing 50 mM potassium phosphate (pH 7.4), 300 mM NaCl, 0.2% CHAPS, 0.2% β-mercaptoethanol, 20% glycerol. 

#### 2.1.2. Cytochrome P450 2J2 (CYP2J2)

*E. coli* strain BL21(DE3) was transformed with recombinant vector pCW-CYP2J2, carrying a sequence of truncated and His-tagged CYP2J2. The bacteria were grown overnight in 10 mL LB broth (with 100 µg/mL ampicillin) at 37 °C. This overnight culture was used to inoculate one liter of fresh TB medium, and cells were grown at 37 °C with shaking at 180 rpm. Protein expression was induced by adding IPTG to a final concentration of 0.5 mM. The culture temperature was reduced to 22 °C following induction and cells cultivated for 48 h were harvested by centrifugation at 3000× *g* for 10 min. The protein was purified on Ni–NTA resin following SEC on HiLoad 26/60 Superdex 200 pg column. The purified protein was stored in buffer (50 mM potassium phosphate buffer (pH 7.4), 300 mM NaCl, 0.2% sodium cholate, 2 mM tris-(2-carboxyethyl) phosphine (TCEP) and 20% glycerol) at −75 °C for further use.

### 2.2. Tissue Lysate Preparation

Rat tissue samples were supplied from the IBMC’s cryobank. Lysates of testis, aorta, lung, heart, liver, and brain rat tissues were obtained using the CelLytic ™ MT Cell Lysis Reagent lysis buffer (Sigma Aldrich, St. Louis, MO, USA) according to the manufacturer’s protocol. Protease inhibitor cocktail (Cat. No. 80-6501-23, GE Healthcare, Boston, MA, USA) was added to lysates. Total protein concentrations in rat tissue lysate samples were determined by the Bradford method. Lysates were aliquoted (500 µL/plastic tube) were stored at −40 °C.

### 2.3. Reagents and Buffers

The following buffer solutions and reagents were obtained from GE Healthcare (USA): Buffer A—HBS-N buffer (150 mM NaCl, 10 mM HEPES, pH 7.4); Buffer B—HBS-EP + buffer (150 mM NaCl, 2 mM EDTA, 0.05% Tween, 10 mM HEPES, pH 7.4) containing 2 mM dithiothreitol (DTT); regeneration solution with 2M NaCl and 0.4% CHAPS; an aqueous solution 0.1 M NHS (N-hydroxysuccinimide) and 0.4 M EDC (1-ethyl-3- (3-dimethylaminopropyl) carbodiimide hydrochloride) and CNBr-activated Sepharose 4B. Other chemicals were obtained from local suppliers. 

### 2.4. Surface Plasmon Resonance (SPR)

#### 2.4.1. Immobilization of the PTGIS Protein on the Optical Chip

All SPR measurements were performed at 25 °C in a four-channel optical biosensor Biacore T-200 (GE Healthcare, USA) operated using the Biacore T-200 Control Software. The biotinylated PTGIS was noncovalently immobilized on the streptavidin-coated chip (SA) in the working channel up to 4000 RU (about 4 ng of a bound protein). Buffer A was used as a running buffer. It should be pointed out that PTGIS preparation was immobilized mainly as monomers because in additional experiments we did not observe the significant biosensor signal decrease under harsh regeneration conditions. 

#### 2.4.2. Comparative Estimation of Probable Fished Protein Material in the Rat Tissue Lysates

Samples of rat tissue lysates were normalized to the total protein concentrations by diluting the initial samples with CelLytic™ MT Cell Lysis Reagent lysis buffer to 2 mg/mL. All SPR measurements were performed in buffer B as a running buffer. Samples of lysates were then diluted with buffer B 40 times and injected for 5 min at a flow rate of 3 μL/min. Regeneration of the chip surface after each injection was carried out with regeneration solution for 30 s at a flow rate of 30 μL/min.

#### 2.4.3. Sample Preparation for Model SPR Experiments

All sample preparation steps were carried out on ice. An aliquot of 100 μL of 100 mM HCl was added to a sample containing 90 μL of 2× buffer A and 10 μL of testis lysate (4 mg/mL of a total protein) to drastically shift the pH value from 7.4 to 2.0 and subsequent incubation on ice for 1 min. Then, 30 μL of NaOH aqueous solution (~300 mM) was added to restore the pH value from 2.0 to 7.4 and 170 μL of 2× buffer B with a protease inhibitor cocktail was added to a final sample volume of 400 μL. 

A lysate sample without acid treatment was used as a control. A sample containing 10 μL of lysate (4 mg/mL) and 190 μL of 1X buffer A was incubated on ice for 1 min. Then, an aliquot of 200 μL of 2× buffer B was added to a final sample volume of 400 μL. In the additional experiments, exogenous PTGIS protein was added in both lysate samples to its final concentration of 50 μg/mL after the pH value had been restored to 7.4 (Table S1, Supplementary File 1). All lysate samples were injected over the optical chip surface with immobilized PTGIS for 1 min at a flow rate of 3 μL/min. Regeneration solution was injected for 30 s at a flow rate of 30 μL/min to regenerate the chip surface after each lysate injection.

To monitor the re-association of protein complexes in the lysate after acid treatment, aliquots were taken from the lysate samples incubated on ice at irregular intervals to determine the levels of protein material binding with the immobilized PTGIS on the optical chip. Lysate samples without acid treatment were used as a control.

### 2.5. Preparation of Control and Affine Sorbent

CNBr-activated Sepharose 4B was used for covalent PTGIS coupling via amino groups of the protein according to the protocol described in [16]. Briefly, 200 μL of immobilization buffer containing 500 mM NaCl, 100 mM NaHCO_3_ (pH 8.3) and PTGIS protein were added to about 200 µL suspension of CNBr—activated Sepharose 4B (100 mg of dry sorbent). After incubation for 2 h at room temperature, inactivation of remaining reactive groups of the sorbent was performed by buffer containing 150 mM NaCl, 100 mM Tris-HCl (pH 7.4) overnight at + 4 °C. The efficiency of protein immobilization was assessed spectrophotometrically by determining the protein concentration before and after immobilization. The average level of protein immobilization was about 3 mg per 1 g of dry sorbent. The control sorbent was prepared similarly with the omitting the protein immobilization step.

### 2.6. Estimation of Specific and Non-Specific Binding for Control and Affine Sorbent

A volume 200 μL of a suspension of the control sorbent (without PTGIS immobilization) or affine sorbent (with immobilized PTGIS) was placed in microcolumn. A volume of 400 μL of acid-treated (or control) lysate samples was incubated for 2 h with sorbents in the presence of protease inhibitors at +4 °C. Then, the protein material weakly or non-specifically bound with the sorbent was washed with buffer B, and the affine-bound proteins with PTGIS were eluted with solutions containing 0.4% CHAPS and NaCl gradient (from 0.25 to 2.0 M). Several eluates (500 µL each) were collected and injected through the working channel with immobilized PTGIS for 3 min at a flow rate of 3 μL/min.

### 2.7. Affinity Isolation of Protein Partners of PTGIS from Testis Tissue Lysate

A preparative variant of direct molecular fishing was carried out using an original microcolumn with a total volume of 200 μL filled with control or affine sorbents, installed in a chromatograph AKTA Purifier 10 (GE Healthcare, USA) operating under UNICORN v5.31 software. The sorbent was equilibrated with running buffer (Buffer B) for 90 min at a flow rate of 50 μL/min. Lysate preparation was performed on ice and is shown in Table S2 (Supplementary File 1). A volume of 4 mL of the prepared lysate sample was passed through the microcolumn at 15 °C for 80 min at a flow rate of 50 μL/min. After washing with running buffer, specifically bound proteins were eluted by regeneration solution for 100 min at a flow rate of 50 μL/min. The total protein content in the eluates, determined by the Bradford method, was about 25–35 µg/mL. The experiments were repeated twice.

### 2.8. Size-Exclusion Chromatography (SEC) Fractionation of Tissue Lysate

SEC fractionation of rat testis tissue lysate was performed on an AKTA Purifier 10 chromatograph (GE Healthcare, USA) thermostated at 15 °C. A lysate sample, diluted with Buffer B up to 2 mg/mL of total protein, was centrifuged at 12,000× *g* for 10 min at 4 °C. The supernatant was collected and then fractionated on a HiLoad 16/600 column with Superdex 200 prep grade (GE Helthcare, USA) at a flow rate of 800 μL/min. 22 lysate fractions (1.5 mL each) were collected, with average molecular weights of 15, 30, 42, 45, 50, 55, 60, 65, 75, 90, 105, 120, 135, 155, 175, 200, 230, 265, 310, 360, 420, 490 kDa. The total protein concentration in the fractions (ranging from 0.6 to 2.5 mg/mL) was estimated based on a calibration curve reflecting the dependence of the area under the chromatographic curve at a wavelength of 280 nm on the total mass of the protein entering the measuring cell of the chromatograph. 

### 2.9. LC/MS-MS Analysis

For LC-MS/MS analysis, a universal method of sample preparation was used, originally proposed [18] and modified by us. It is based on the use of a centrifuge tube concentrator Vivaspin 500 with MWCO 10 kDa (Sartorius, Goettingen, Germany) in which all the procedures are performed: removal of detergents and salts; SDS protein denaturation; partial proteolysis by trypsin and separation of peptide fragments from residual high molecular weight components of the sample. For tryptic cleavage samples containing 30 µg of total protein were used.

### 2.10. Liquid Chromatography

Peptides were separated using liquid chromatography on an Ultimate 3000 RSLC Nano (Thermo Scientific, Waltham, MA, USA) system equipped with 5 µL loop (loop flush out multiplier—3). Samples were thermostated in the autosampler at 81 °C. Samples were drawn at 12 µL/min and injected into the loop at 15 µL/min. Samples were loaded onto an enrichment column Acclaim Pepmap® (5 mm × 0.3 mm, 300 Å pore size, 5 µm particle size) for 4 min at a flow rate of 15 µL/min in a mobile phase C (water with 3.5% acetonitrile supplied with 0.1% formic acid and 0.05% acetic acid (pH 2.75) and at 21 °C). Peptides were washed out from the enrichment column and separated into the analytical column Acclaim Pepmap® (75 µm × 150 mm, 1.8 µm particle size, 60 Å pore size) at a flow rate of 0.30 µL/min in a gradient of mobile phase A (water, pH 2.63, and at 21.4 °C) and mobile phase B (90% acetonitrile and 10% methanol), both supplied with 0.1% formic acid and 0.03% acetic acid. A dynamic flow rate from 0.30 to 0.45 µL/min within 1.25 min was applied for column washing in mobile phase B for 6 min. 

### 2.11. Mass Spectrometry

Mass spectrometry analysis was performed on a high-resolution Orbitrap Fusion (Thermo Scientific, USA) mass spectrometer. The instrument was equipped with an NSI ion source and analyzed in a positive ionization mode using a three-segmental data-dependent survey. Apart from the commonly used conditions of quadrupole isolation with (±1.5 Th) with asymmetric offset (+0.5 Th), normalized resolution for MS1 scans (R = 60K), electrodynamic S-lens adjustment at 70% and capillary vaporizing temperature set to 280 °C, other parameters were adjusted and installed specifically for each segment. 

Segment A: segment A was focused on detection of small and poorly ionized peptides that might typically interfere with low molecular weight compounds still presented in the sample even after desalting. Precursor ions were surveyed at a resolution of R = 60K in a range of 400–650 m/z for a maximum integration time of 10 ms, or AGC (acquisition gain control) accumulation of 2e5 ions. Ions were isolated using quadrupole within ±1 Th isolation width with +0.25 Th offset. Tandem MS/MS scanning in a linear ion trap detector was harmonized using isotope-ration trigger mode for ions of z = 1+…2+ charge states with isotope ratio of ΔM = 1.0033 u and isotope ration of 0.54 ± 9% with asymmetric tolerance of +7/−15 ppm. Ions were fragmented using CID mode with a maximum integration time of 35 ms, or AGC control of 1e4 ions, at a 38% of normalized activation energy. Dynamic exclusion for 90 s after 7 repeats within 3 s was activated. The actual analytical time for segment A lasted from the 4th to the 25th minute, and the duty time of the full cycle was set at 2.5 s.

Segment B: was suitable for detection of conventional peptides that typically represented in a range of charge states of z = 2+…6+ and eluted with LC-gradient. Precursor ions were surveyed at a resolution of R = 60K in a range of 425–1250 m/z. Ions were isolated using quadrupole with ±1.5 Th and +0.5 Th offset isolation window and a maximum integration time of 15 ms, or AGC set to 4e5 ions. MS/MS for precursor ions was triggered at a level of 45% of chromatography peak apex (pre-set average FWHM was 24 s) if minimal SNR was at least 1500 counts. Fragment ions were obtained at HCD activation energy normalized to 27% ramped within ±20% and detected in an ultra high-field orbital mass analyzer at a resolution of R = 15K. Ions were accumulated for a maximum integration time of 47 ms or AGC set to 5e4 ions. If 6 repeats (within 4 s) were recorded for all parallel charge states of the injected precursor ions active dynamic exclusion was for 180 s. The complete duty cycle time was 4 sec and segment B lasted from the 10th to the 50th min of the eluting gradient.

Segment C: segment C was oriented towards the detection of high-mass peptides with multiple charge states. Precursor ions of z = 5+…9+ charge states were surveyed at a resolution of R = 60K in a range of 750–1900 m/z. Ions were isolated using linear ion trap and accumulated for a maximum integration time of 120 ms, or AGC set to 2e5 ions. Tandem MS/MS scanning was accomplished at HCD activation mode normalized at 38% alternating within ±20% range. Fragment ions were detected in an orbital mass analyzer at a resolution of R = 15K with a maximum integration time of 85 ms, or AGC set to 5e4 ions. Active dynamic exclusion for 120 s after 3 repeats within 6 s was employed during analysis. The duty time for a single complete scan cycle was 3 s, and the actual analytical time for segment C lasted from the 37th to the 57th minutes. 

### 2.12. Data Analysis

Vendors’ original data files (raw-format) were converted to fit-for-searching mgf-format using MSConvert (Proteome Wizard). Data were searched using the X!Tandem engine against a taxonomy-specific database (Uniprot version 2018.08) enriched with decoy reversed sequences. The pre-installed search parameters considered trypsin to be a digestion enzyme with 2 maximum internal missed cleavages. The allowed charge states were from z = 1+ to z = 9+ with a precursor tolerance of ±5 ppm and fragment ions tolerance of ±0.01 Da. The variable modifications used for searching and discovery were deamidation of Q/E, methionine single oxidation, and 4-hydroxyproline. The fixed modification was pyridilethylation by 4-vinylpyridine. Results were extracted at no more than 1% of the FDR level based on summarized false discovery rate for PSM, peptides, and proteins with dynamic correction of the recovered sequence results with a molecular weight of the canonical protein sequence. 

### 2.13. Design and Synthesis of Peptides

Molecular docking simulations in the Zdock server [19] and alanine scanning in the Robetta server [20] were performed using PDB files of PTGIS (PDB ID 2iag) and a set of its protein partners isolated from lysate protein partners: GLRX3 (PDB ID 3zyw), HINT2 (PDB ID 4inc), AKR1A1 (PDB ID 4gac), GSTA1 (PDB ID 1k3y), GSTM1 (PDB ID 1xw6), TF (PDB ID 2hau). DockPrep and Minimize structure (300 steps) tools under USCF Chimera [21] were used for preparing files for molecular docking by adding hydrogens and charges and also by removing water molecules. The peptide fragments of protein partners with the predicted hot amino acid residues that are critical for complex formation between PTGIS and a protein partner were selected (Table 1). 

Synthesis of peptides was performed using SPPS technique with a Fmoc/tBu strategy on the ResPepSL automated peptide synthesizer (INTAVIS Bioanalytical instruments, Koeln, Germany). Synthesis scale was 5 μM, the resin of choice was Rink amide (12.5 mg for each reaction vessel). All the required solutions and reagents were prepared in the following concentrations: HBTU 0.5 M, NMM 44% (v/v), NMP 100% (v/v), DCM 100% (v/v), acetic anhydride 5% (v/v), piperidine 20% (v/v), amino acids 0.5 M. Double washing of the column was carried out using DMF to remove unreacted substances, and with DCM to remove the rest of DMF, which can act as a basic reagent and interrupt the acidolysis by TFA during cleavage.

Removal of the peptides from the resin was performed by Cleavage Cocktail treatment. Its composition depended upon the amino acid sequence. The Cleavage Cocktail A containing TFA—95%, TIPS—2.5%, H_2_O—2.5% was used for peptides No. 1, 2, 5−8, 10−12; and Cocktail B containing TFA—90%, TIPS—2.5%, TES—2.5%, H_2_O—2.5%, DTT—2.5% was used for peptides No. 3, 4, 9). Aliquots of the required volume were added to the resin containing anchored peptide and thoroughly mixed within first 5 min. Then it was mixed every 30 min to ensure the reaction was completed. Deprotection occurred within approximately 3.5 h; thereafter, the probes were filtered using PTFE 0.2 μm pore syringe filters (MACHEREY-NAGEL, Duren, Germany).

Peptides were extracted from the solution by precipitation using ice-cold MTBE: at least threefold excess of MTBE was added to the probe followed by centrifugation at 4000 rpm, 4 °C during 10 min on Allegra X15R (Beckman Coulter, Atlanta, GA, USA). The supernatant was removed by decantation. For complete product separation, the procedure including treatment by ether, centrifugation, and decantation was repeated 3 times for each probe.

Precipitated peptides were dissolved in the acetonitrile/H_2_O (1:1) mixture. Solutions were evaporated using the ScanVac centrifugal concentrator (Labogene, Allerod, Denmark) as follows: 1500 rpm, 60 °C, 24 h, vapor trap at −107 °C. The end-product represented a porous white powder. The presence of the targeted peptide was confirmed by MALDI mass spectrometry on Bruker Microflex LFR (Bruker Corporation, Billerica, MA, USA). A probe of approximately 1 mg/mL concentration in ACN/H_2_O was prepared. 0.1% formic acid solution was used as a matrix. 

## 3. Results and Discussion

### 3.1. Model Experiments for Optimization of the Direct Molecular Fishing Procedure

SPR biosensor makes it possible to perform the preliminary semi-quantitative characterization of tissue lysates according to either the presence or absence of protein partners for a bait protein [22,23]. Comparison of different rat tissue lysates (testis, lung, liver, heart, aorta, brain) revealed that the highest binding levels and moderate relative dissociation of the protein material bound from the lysates with immobilized PTGIS were in case of the testis lysate (Table 2). Therefore, further experiments were carried out using testis tissue lysate.

**Figure 1:**
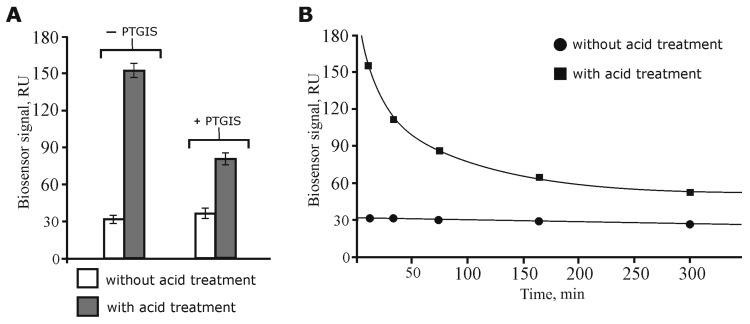
Optical biosensor data of lysate proteins interactions with immobilized PTGIS on the chip: (**A**) SPR analysis of protein binding from diluted lysate samples in the absence or presence of exogenously added PTGIS protein (average mean ± SD, n = 3). (**B**) Model experiments on re-association of protein complexes after acid treatment of lysate sample. The plot of binding levels of protein material from diluted lysate samples versus time of sample incubation on ice; f(x) = 362.77x^−0.34^, *R*^2^ = 0.99.

Recently, we optimized the lysate sample preparation to increase the efficiency of isolation of protein partners from the lysate by carrying out the preliminary dissociation of protein complexes by a short-term acid treatment followed by NaOH neutralization to restore the initial pH value of the samples [24]. In the present study, we additionally compared the SPR binding levels of the protein material from intact (without acid treatment) and acid treated lysates with immobilized PTGIS on the optical chip. It can be seen from Figure 1A that after acid dissociation of the lysate protein complexes, a five-fold increase in the binding level occurred in comparison with intact lysate sample. Furthermore, the addition of exogenous PTGIS to the acid-dissociated and neutralized lysate sample resulted in almost 50% reduction in the binding levels (the biosensor signal decreased from 155 to 80 RU, Figure 1A) of protein material to PTGIS immobilized on the chip as compared to the control lysate (without the addition of exogenous PTGIS). Control SPR experiments did not show any significant binding differences between immobilized PTGIS on the optical chip with itself injected as analyte (oligomerization control). Thus, these experiments have demonstrated that: (1) acid dissociation does indeed cause the release of protein partners of PTGIS from the “wild-type” protein complexes in the lysate and promote an increase in their local concentration; (2) the released protein partners from protein complexes were specifically titrated by addition of exogenous PTGIS.

Another aspect concerns model experiments with the determination of the time scale of the reassociation rate for lysate proteins exposed to the acid treatment. Figure 1B shows that with prolonged incubation on ice of the acid treated and then neutralized lysate samples (without the addition of exogenous PTGIS), the binding of the protein material to PTGIS immobilized on the chip decreased. We suggest that this was due to the slow reassociation of protein complexes, including proteins which could bind to PTGIS. The data indicate that the dissociation of the wild-type lysate protein complexes was indeed achieved, with about half of them being reassociated in the lysate during the first hour of incubation. Maintenance of stable biosensor binding signals, after injection of preincubated control lysate samples (without acid treatment), suggests that formation of the protein complexes during a period of incubation did not change significantly (Figure 1B).

The following model experiments made it possible to assess the specificity of the molecular fishing procedure when applied to the inert sorbent used. The PTGIS protein was covalently immobilized on the activated CNBr-sepharose 4B via amino groups of the protein. A similar protocol has already been used to immobilize recombinant thromboxane synthase [16] and some recombinant proteins encoded by genes on Chromosome 18 [12,14,25] on this sorbent. The specificity of direct molecular fishing was analyzed by SPR. Eluates containing bound lysate protein material from the empty (control) and affinity (with PTGIS) sorbents were injected over the chip surface with immobilized PTGIS. Table 3 shows that a total 4.5-fold excess of the binding level of the protein material being eluted by a NaCl gradient from the affinity sorbent, as compared to the control one, may indicate that the eluted proteins formed specific stable complexes with PTGIS on the chip. It should be noted that the SPR analysis of samples containing weakly bound proteins eluted by running buffer from both control and affinity sorbents did not reveal any differences in the biosensor signals (Table 3). Since it was found that the highest elution efficiency of the protein material from the affinity sorbent occurred when using an eluent containing 2M NaCl and 0.4% CHAPS; therefore, it was used in the preparative molecular fishing procedure.

### 3.2. Isolation and Identification of Potential Protein Partners for PTGIS from Testis Tissue Lysate by Affinity Chromatography and LC-MS/MS

The general scheme of the experiments performed according to the preparative version of the direct molecular fishing procedure can be summarized as follows. The PTGIS protein was covalently immobilized on to the CNBr-activated Sepharose 4B (affine sorbent) via surface amino group of the protein. Lysate samples, in which the protein complexes had been previously dissociated by short-term acid treatment and then neutralized to the initial pH value, were passed through the control or affine sorbent. We assumed that a portion of PTGIS’s protein partners in the wild-type protein complexes in the lysate should become capable of interacting with PTGIS immobilized on the affine sorbent after acid treatment of lysate. On the other hand, acid treatment of the lysate would increase the efficiency of the direct molecular fishing procedure by reducing the number of secondary protein partners that can be co-isolated as a component of the multiprotein complexes “tagged” with direct protein partner of PTGIS. The specificity of the isolation of PTGIS’s protein partners from the lysate was estimated both by using the control and the affine sorbents with the intact lysate.

**Figure 2:**
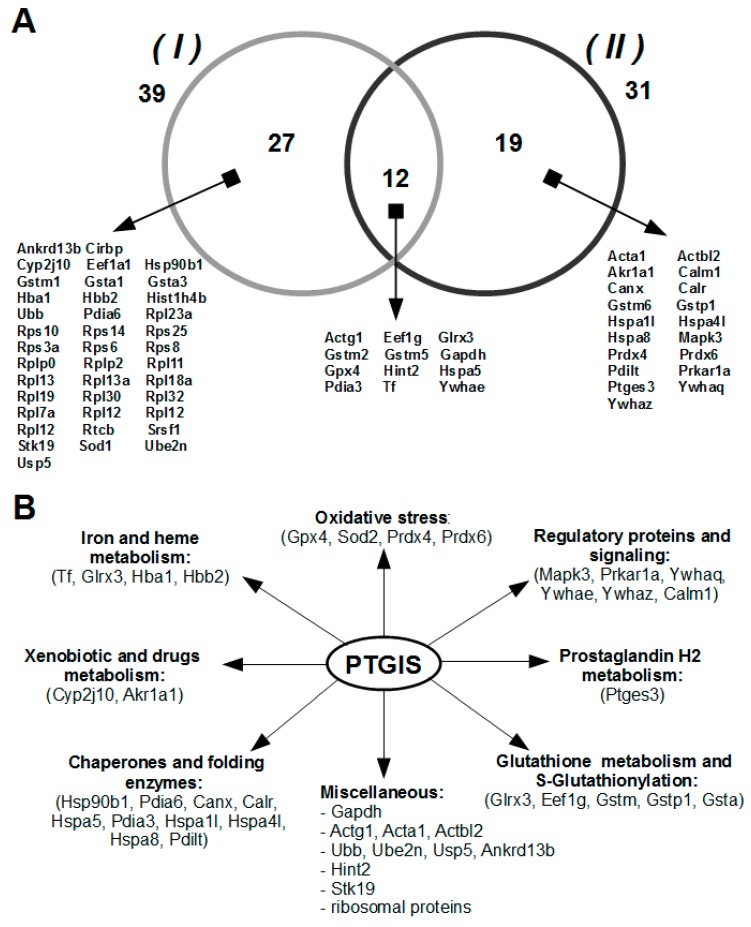
Proteins isolated and identified from the lysate by direct molecular fishing procedure using PTGIS as a bait protein: (**A**) Final listing of potential protein partners of PTGIS isolated from intact (I) and acid-treated (II) testis tissue lysate using affine sorbent (with immobilized PTGIS protein) by excluding proteins non-specifically bound to empty sorbent (without PTGIS). (**B**) The main functional roles of potential protein partners of PTGIS.

The lists of potential protein partners of PTGIS identified by LC-MS/MS in eluates from the affine column are presented in Supplementary File 2. It should be noted that these lists were corrected for proteins nonspecifically bound to the empty sorbent (without PTGIS). Figure 2A demonstrates distribution of protein partners isolated from the intact and acid-treated lysates. It is clear that 58 unique proteins were identified by LC-MS/MS, twelve of which were isolated from both lysates used. Therefore, we believe that the comparison of the two lists of identified proteins can give a much better picture of the whole possible spectrum of protein partners for prostacyclin synthase. The functional diversity of PTGIS protein partners is shown in Figure 2B. A large number of isolated from lysates potential protein partners that belong to different functional subgroups may indicate involvement of PTGIS in other molecular processes than those documented on the basis of current level of understanding of the functional characterization of potential protein partners of PTGIS. 

### 3.3. Functional Intersection between PTGIS and Identified Protein Partners

Prostaglandin synthase (PTGES) and 14-3-3 epsilon isolated from the testis tissue lysate were consistent with the actual information on protein partners of prostacyclin synthase in the interactome databases (Funcoup, String and Mint). Furthermore, we considered some identified protein partners of PTGIS in terms of the presence of any connections between them and PTGIS. AKR1A1 protein is involved in the conversion of endogenous and exogenous metabolites and synthesis of prostaglandins and steroidal hormones [26]. Prostaglandin synthase enzymes were known to belong to the superfamily of glutathione S-transferases and require glutathione as an essential co-factor for activity; they also show significant homology with other members of the MAPEG superfamily involved in eicosanoid and glutathione metabolism [27,28,29,30]. Direct participation of GSTs in prostaglandin A_2_ and prostaglandin J_2_ conjugation with glutathione was shown by in vitro experiments [31]. The maintenance of protein structure in the cluster is perhaps provided by molecular chaperones HSP90B1 and HSPA5, as well as by Pdilt and PDI proteins [32,33], as these proteins were also isolated from lysates on the affine column. Although there is no reason to regard glyceraldehyde 3-phosphate dehydrogenase (GAPDH, EC 1.2.1.12) as a direct protein partner, certain evidence exists that GAPDH and other glycolytic enzymes (ENO2 and PKM1) are target proteins for the prostaglandin 15d-PGJ_2_ [34]. Thus, the presence of GAPDH among prey proteins can be attributed to its isolation from the lysate as a part of some multiprotein complex.

Prostacyclin synthase is quite distantly related to cytochrome P450s superfamily according to 20-25% identity of the amino acid sequences. The most frequent cytochrome, P450’s, post-translational modification is phosphorylation, which is found in about 30 sources (see the review [35]). Isolated 14-3-3 proteins, as potential protein partners of PTGIS, can recognize the canonical phosphorylated motifs of PTGIS protein in predicted amino acid positions 24–36, 75–82, 200–206, 223–232 using the online server [36] (http://www.compbio.dundee.ac.uk/1433pred). Other ways of post-translational regulation of prostacyclin synthase activity have been described to occur via reversible or irreversible S-nitrosylation [35,37], as well as S-glutathionylation of Cys residues of PTGIS protein [38]. Thus, it can be speculated that isolation from testis tissue lysate of multifunctional protein glutaredoxin (GLRX) [39,40,41,42], as a potential protein partner of PTGIS, indicates a probable role of GLRX in the S-glutathionylation of PTGIS. 

### 3.4. SEC Profiling of Tissue Lysate Proteins

Tissue lysates are experimental models containing all types of cells that can form a tissue, providing important information about the pattern of participation of a particular cell protein in the formation of stable protein complexes, and SEC profiling of tissue lysates is another tool for the disclosure of such information [12,22,23,25]. SEC profiling of lysate made it possible to expand molecular fishing information about identified proteins. Thus, it hints at possible ways of complex formations of protein partners with PTGIS. The testis tissue lysate sample was separated into several fractions corresponding to different molecular weights, and the distribution of identified protein partners of PTGIS among the lysate fractions are shown in Supplementary File 3. Then, the proteins were combined into five groups (Table 4) depending on the pattern of their participation in complex formation in the intact lysate sample. From Table 4, it follows that about 50% of identified protein partners of PTGIS are in monomeric form (groups I, II, IV; see Table 4), and therefore are potentially accessible for affinity interaction with the bait proteins, for example, PTGES3, Gpx4, Sod1, Gstm1, Gstm2, Gapdh, TF and others. However, Ywhaq, Ywhaz, Canx, Prkar1a, Hspa1l, Hspa8, Prdx4 proteins were present only in high molecular weight fractions (group V, see Table 4), and were identified in the eluates from the affinity sorbent only after acid treatment of the lysate. It appears that they become potentially accessible for interactions with PTGIS as a bait protein only after release from the wild-type stable lysate protein complexes due to acid dissociation.

Among the proteins identified in the intact lysate, there are those that form large protein complexes involved in universal molecular processes such as translation and splicing of proteins (ribosomal proteins; RNA-binding proteins (Cirbp, Rtcb); elongation factors (Eef1a1); ubiquitination proteins (Ube2n and Usp5). Fragments of multiprotein molecular complexes, consisting of some identified proteins, can be demonstrated in the following examples: (Pdia6—HSP90b1—HSPA5) [43]; (HSP90b1—HSPA5—Ube2n) [ID 6859, CORUM database, http://mips.helmholtz-muenchen.de/corum/]; (GAPDH—Pdia3) [ID 280, CORUM database]. However, it is still difficult to discuss any involvement of PTGIS as an integral part of these protein complexes.

### 3.5. Partial SPR Validation of Protein–Protein and Protein–Peptide Interactions between PTGIS and Its Potential Protein Partners

Cytochrome P450 2J10 (CYP2J10) was identified as a potential protein partner for PTGIS protein (Figure 2A) from intact testis tissue lysate. The recombinant protein CYP2J2, sharing 70% sequence identity with rat CYP2J10, was used for SPR validation. It was determined that the dissociation constant for PTGIS/CYP2J2 complex was 200 nM (Figure S1; see Supplementary File 1). As for the functional consequences of the complex formation of PTGIS/CYP2J2, we can assume the possible involvement of these proteins in the metabolism of arachidonic acid [44] and prostacyclin as a P450-dependent epoxygenase [45].

Glutathione S-transferase (GST) isoforms differ from each other by virtue of the primary amino acid sequence (25–75% identity) and exhibit different catalytic properties, but mainly exist in the form of homodimers with a molecular weight of 44–50 kDa. The dissociation constant of the GST homodimer, previously determined by several methods, varied in a wide concentration range (from 10^−6^ M to 10^−9^ M) [46,47,48,49]. However, GST may also exist in the monomeric form [50,51], which is catalytically active [46,49]. According to the SEC profiling of the testis tissue lysate (see Supplementary File 3 and Table 4), GST isoforms (GSTA1, GSTM1, GSTM2, GSTM5) isolated from the tissue lysate on affine sorbent with PTGIS, were detected in the lysate fractions with a molecular weight of 30–50 kDa (i.e., in monomeric or dimeric form), and hence were potentially capable of binding with PTGIS. Thus, the purpose of SPR validation was to investigate the interactions of PTGIS with GST under standard conditions. The GST preparation (Cat. No. G6511, Sigma Aldrich) was immobilized on the CM5 optical chip via the amino groups of the protein, but there was no interaction with PTGIS injected as an analyte in concentrations up to 25 µM. Interestingly, after short-term pH-dependent dissociation of immobilized GST into monomers which resulted from an exact two-fold stepwise drop in the biosensor signal, the binding of PTGIS was recorded in a concentration range of 10^−7^ to 10^−6^ M (Figure S2; see Supplementary File 1). It should also be said that there was no binding in the case of the SPR analysis of an inverted protein pair, i.e., when PTGIS was immobilized on the optical chip, and GST preparations were injected as an analyte.

**Figure 3:**
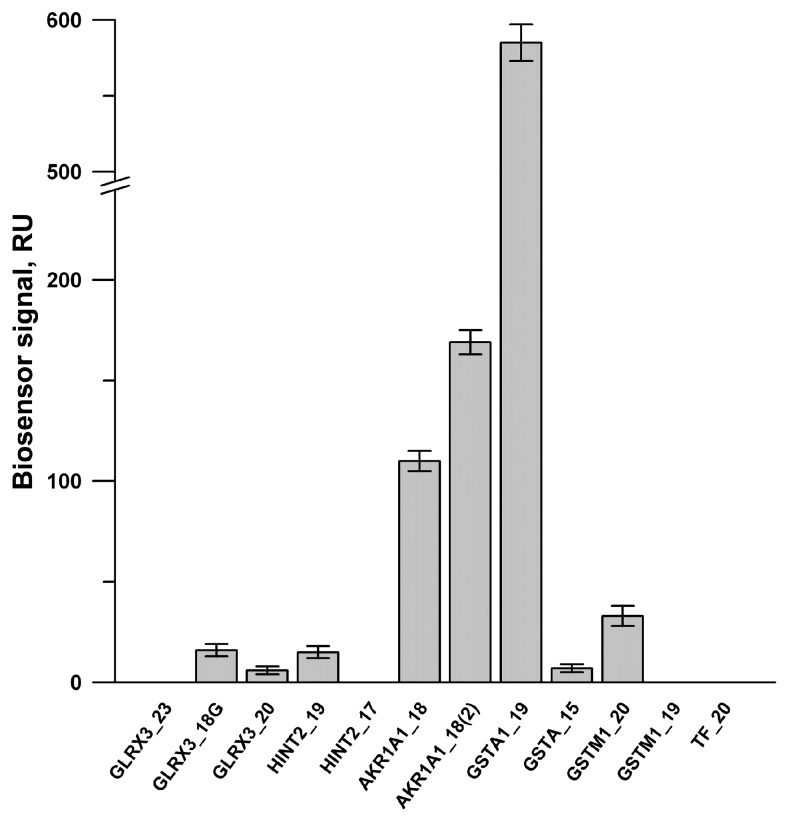
The diagram of average peptide binding levels ± SD (n = 3) with immobilized PTGIS protein. PTGIS was covalently immobilized on to the CM3 chip via amino groups of a protein up to 4000 RU (or 4 ng of a protein per 1 mm^2^ of chip surface). HBS-N was used as a running buffer. Peptide samples (20 μM) were injected for 7 min at a flow rate of 10 μL/min. Regeneration of the chip surface after each peptide injection was performed with a solution containing 2 M NaCl and 0.4% CHAPS for 30 s at a flow rate 30 µL/min.

SPR screening of the binding capability of twelve peptides synthesized with immobilized PTGIS on the optical chip is presented in Figure 3. It was shown that positive binding signals were observed for the peptides derived from GSTA1, AKR1A1, GLRX3, GSTM1, HINT2 (Figure 3). Further SPR experiments confirmed that the peptide binding with PTGIS in a concentration-dependent manner was observed only for four peptides GSTA1_19, AKR1A1_18, AKR1A1_18 (2) and GLRX3_18. Apparent Kd values of PTGIS/peptide complexes were calculated in the range of 10^−5^ M–10^−6^ M (Figure S3–S6; see Supplementary File 1). Using the jsPISA 2.0.5 program (http://www.ccp4.ac.uk/pisa/) [52] for structural analysis of protein–protein interfaces of GSTA1 (PDB ID 1k3y), we found that peptide GSTA1_19 ^67^QTRAILNYIASKYNLYGKD^86^ contains amino acid residues that are critical for the interface contacts of two GSTA1 monomers (Table S3, see Supplementary File 1). In contrast, peptide GSTA1_15 ^208^MDEKSLEEARKIFRF^222^ did not contain any amino acid residues involved in the homodimerization of GSTA1 protein. SPR analysis revealed that only “interfacial” peptide GSTA1_19 interacted with PTGIS (Figure S3, see Supplementary File 1). Thus, we have shown for the first time that prostacyclin synthase, can form a heterocomplex with the monomeric form of glutathione S-transferase, presumably due to the involvement of amino acid residues in the GST/GST homodimerization interface, whereas PTGIS does not interact with the dimeric form of GST.

### 3.6. The Sensitivity of PTGIS to Small Compound Isatin (indole 2,3-dion)

**Figure 4:**
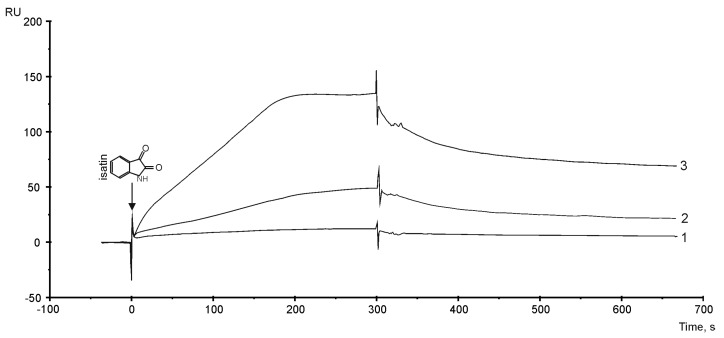
SPR sensorgrams of isatin interaction with PTGIS immobilized on the SA chip using the streptavidin–biotin coupling protocol. Biosensor signals represent the difference between working and control channels. About 9000 RU (approximately 9 ng) of biotinylated PTGIS was immobilized on the SA chip. The following concentration of isatin were injected for 5 min at a flow rate of 10 µL/mL: (1) −43 µM, (2) −86 µM, (3) 129 µM. HBS-EP+ buffer was used as a running buffer.

Recently, we found that the non-peptide endogenous bioregulator isatin (Mw = 147 Da) bound some cytochrome P450s isoenzymes and influenced the affinity of protein–protein interactions [16,53,54]. Therefore, to continue this investigation, SPR validation of the isatin binding (injected as analyte) with PTGIS protein immobilized on the optical chip was performed. It was noticed that there was a positive concentration-dependent binding of isatin with PTGIS immobilized on the optical chip, but with a stoichiometry of more than 1:1 at concentrations above 43 μM (Figure 4). The theoretical isatin binding capacity of the immobilization level of PTGIS (assuming a 1:1 binding stoichiometry and 100% ligand activity) corresponds to 28 RU. Thus, at the saturating concentration of isatin (129 μM), at least four possible isatin binding sites can be expected on the PTGIS surface. The range of interacting isatin concentrations with PTGIS generally corresponds to the physiological range of this compound in the tissues of living organisms (0.1–100 μM) [53]. Thus, PTGIS protein represents a new direct target for the action of bioregulator isatin, which can potentially affect protein activity and modulate the affinity of PTGIS’s interactions with other protein partners.

## 4. Conclusions

In living organisms, most proteins function not only as part of binary protein complexes, but also represent a higher level of organization, i.e., multimeric protein complexes. In the current work, using the methods of classical proteomics, the authors isolated from rat testis tissue lysate the new potential protein partners for prostacyclin synthase (PTGIS) and then identified them by LC/MS-MS. The relevance of the optimized experimental conditions, effectiveness and specificity of the preparative affine isolation of proteins were demonstrated. Also, in vitro biophysical system based on the surface plasmon resonance technology allowed to characterize several binary protein–protein and protein-peptide interactions which, theoretically, may not have biochemical significance as dimers, but they are probably involved in multimeric protein complexes with PTGIS. In part, biochemical significance can be indirectly verified by comparing data on the intracellular localization of the bait/prey proteins forming a complex and their annotated functions, but the final answer on the functionality of a particular binary interaction with PTGIS can indeed be obtained from in vitro biochemical tests with estimation of prostacyclin production, which are planned to be conducted in the future. 

## Figures and Tables

**Table 1:** Listing of synthesized peptides to identified potential protein partners of prostacyclin synthase (PTGIS).

No	Mw, Da	Name	Sequence	Purity by HPLC, %
1	2640	GLRX3_23	K-E-L-E-A-S-E-E-L-D-T-I-C-P-K-A-A-E-N-L-Y-F-Q	>90
2	2597	GSTM1_20	D-Y-D-R-S-Q-W-L-N-E-K-F-K-L-G-L-D-F-P-N-L	>90
3	2437	TF-20	T-D-F-P-Q-L-C-Q-L-C-P-G-C-G-C-S-T-L-N-Q-Y-F	>90
4	2284	GSTM1_19	N-Q-T-M-D-N-H-M-Q-L-G-M-I-C-Y-N-P-E-F	>95
5	2278	GLRX3_20	D-I-V-K-E-L-K-E-N-G-E-L-L-P-I-L-R-G-E-N	>90
6	2230	GSTA1_19	Q-T-R-A-I-L-N-Y-I-A-S-K-Y-N-L-Y-G-K-D	>90
7	2141	AkrA1_18(2)	K-R-V-P-R-D-A-G-H-P-L-Y-P-F-N-D-P-Y	>95
8	2042	HINT2_17	S-V-Y-H-L-H-I-H-V-L-G-G-R-Q-L-Q-W	>90
9	2202	AkrA1_18	K-Q-L-D-A-L-N-K-N-W-R-Y-I-V-P-M-I-T	>90
10	1976	HINT2_19	T-A-K-A-E-G-L-G-D-G-Y-R-L-V-I-N-D-G-K	>90
11	1961	GLRX3_18	E-V-G-S-A-G-Q-F-E-E-L-L-R-L-K-A-K-S	>90
12	1897	GSTA1_15	M-D-E-K-S-L-E-E-A-R-K-I-F-R-F	>90

**Table 2:** Binding levels of protein material from rat tissue lysates with prostacyclin synthase immobilized on the optical chip.

Tissue Lysates	Biosensor Signal after Sample Injection, RU *			Dissociation Rate, RU/min	Relative Dissociation, %
-	After 30 s	After 360 s	Δ300 s	-	-
Testis	126 ± 4 **	88 ± 4	38	8	30%
Lung	74 ± 3	56 ± 3	18	4	24%
Liver	65 ± 3	52 ± 3	13	3	20%
Heart	51 ± 3	38 ± 2	13	3	25%
Aorta	48 ± 2	26 ± 2	22	5	46%
Brain	32 ± 2	19 ± 2	13	3	41%

* RU (resonance unit) corresponds to 1 pg of protein bound to 1 mm ^2^ of the chip surface. ** Average binding levels, mean ± SD, n = 4.

**Table 3:** SPR analysis of samples containing protein material washed and eluted from empty and affine sorbents after lysate incubation.

Buffer Type	Volume, mL	Volume (on a Cumulative Basis), mL	Empty Sorbent (without PTGIS Immobilization)	Affine Sorbent (with PTGIS)
Wash buffer			Average binding levels (n = 2) with PTGIS on the chip, RU *	
1X HBS-EP+	1	1.0	67	73
1X HBS-EP+	1	2.0	58	48
1X HBS-EP+	1	3.0	17	23
1X HBS-EP+	1	4.0	3	8
1X HBS-EP+	1	5.0	0	5
		Total	145	157
Elution buffer				
0.25 M NaCl and 0.4% CHAPS	0.5	5.5	0	0
0.5 M NaCl and 0.4% CHAPS	0.5	6.0	0	7
0.75 M NaCl and 0.4% CHAPS	0.5	6.5	0	8
1.0 M NaCl and 0.4% CHAPS	0.5	7.0	1	13
1.5 M NaCl and 0.4% CHAPS	0.5	7.5	1	10
2.0 M NaCl and 0.4% CHAPS	0.5	8.0	8	26
		Total	10	54

* 1 RU (resonance unit) corresponds to 1 pg of protein bound to 1 mm^2^ of the chip surface.

**Table 4:** Analysis of testis tissue lysate fraction distribution (according to SEC profiling) of isolated potential protein partners of PTGIS.

Protein Name	Gene Name	Subunit Structure *	Groups of Proteins ** (According to SEC Profiling of Lysate)				
			I	II	III	IV	V
PROTEIN PARTNERS ISOLATED FROM INTACT LYSATE ONLY							
Cold-inducible RNA-binding protein	Cirbp						■
Cytochrome P450, family 2, subfamily j, polypeptide 10	Cyp2j10						■
Elongation factor 1-alpha 1	Eef1a1					■	
Endoplasmin	Hsp90b1						■
Glutathione S-transferase	Gstm1	Homodimer		■			
Glutathione S-transferase alpha-1	Gsta1	Homodimer			■		
Glutathione S-transferase alpha-3	Gsta3	Homodimer		■			
Hemoglobin subunit alpha-1/2	Hba1					■	
Hemoglobin subunit beta-2	Hbb2					■	
Histone H4	Hist1h4b	Heterooligomer					■
Protein disulfide-isomerase A6	Pdia6						■
Superoxide dismutase [Cu-Zn]	Sod1	Homodimer		■			
tRNA-splicing ligase RtcB homolog	Rtcb						■
Ubiquitin-conjugating enzyme E2 N	Ube2n	Heterodimer		■			
PROTEIN PARTNERS ISOLATED FROM ACID TREATED LYSATE ONLY							
14-3-3 protein theta	Ywhaq	Homodimer			■		■
14-3-3 protein zeta/delta	Ywhaz	Homodimer			■		■
Actin, alpha skeletal muscle	Acta1			■			
Alcohol dehydrogenase [NADP(+)]	Akr1a1					■	
Calmodulin-1	Calm1				■		
Calnexin	Canx						■
Calreticulin	Calr					■	
cAMP-dependent protein kinase type I-alpha regulatory subunit	Prkar1a						■
Heat shock 70 kDa protein 1-like	Hspa1l						■
Heat shock cognate 71 kDa protein	Hspa8						■
Peroxiredoxin-4	Prdx4	Homodimer					■
Peroxiredoxin-6	Prdx6	Homodimer				■	
Protein disulfide-isomerase-like protein of the testis	Pdilt	Homodimer			■		
Ptges3 protein	Ptges3			■			
PROTEIN PARTNERS ISOLATED FROM BOTH INTACT AND ACID TREATED LYSATES							
14-3-3 protein epsilon	Ywhae	Homodimer			■		■
78 kDa glucose-regulated protein	Hspa5						■
Actin, cytoplasmic 2	Actg1					■	
Elongation factor 1-gamma	Eef1g						■
Glutathione peroxidase	Gpx4		■				
Glutathione S-transferase	Gstm2	Homodimer				■	
Glutathione S-transferase Mu 5	Gstm5	Homodimer				■	
Glyceraldehyde-3-phosphate dehydrogenase	Gapdh	Homotetramer				■	
Protein disulfide-isomerase	Pdia3			■			
Serotransferrin	Tf					■	

* from UniProt database; ** conditional groups of proteins depending on their lysate position according to SEC profiling data (see also Supplementary File 3): I group—proteins are in monomeric state only (protein Mw = lysate fraction Mw); II group—proteins are in monomeric and homo-, or heterodimeric state; III group—proteins are in homo-, heterodimeric state only; IV group—proteins are in monomeric state and in high molecular weight complexes; V group—proteins are in high molecular weight complexes only.

## Supplementary Materials

The following are available online at https://www.mdpi.com/2079-7737/8/2/49/s1; Supplementary File 1 contains following information: Table S1: (Sample preparation of lysate for model SPR experiments), Table S2: (Sample preparation of lysate for affinity isolation of protein partners of prostacyclin synthase), Table S3: (amino acid residues participating in GSTA1–GSTA1 interactions), Figure S1: (SPR sensorgrams of interaction between PTGIS and CYP2J2), Figure S2: (SPR sensorgrams of interaction between GST and PTGIS), Figure S3: (SPR sensorgrams of interaction between PTGIS and peptide from GSTA1, Figure S4: (SPR sensorgrams of interaction between PTGIS and peptide from GLRX3, Figure S5 and S6: (SPR sensorgrams of interaction between PTGIS and peptides from AKR1A1, Supplementary File 2: contains a list of proteins identified by LC-MS/MS with added data on intracellular localization from Uniprot and GenCards databases, Supplementary File 3: contains information on position of PTGIS’s protein partners in SEC fractions of lysate.

## Abbreviations

PTGIS	Prostacyclin synthase
TBXAS1	thromboxane synthase
PGH_2_	prostaglandin endoperoxide H2
PGE_2_	prostaglandin E2
Kd	dissociation constant of molecular complex
ACN	acetonitrile
DCM	dichloromethane
DMF	*N,N*-dimethylformamide
DTT	dithiothreitol
HBTU	3-[bis(dimethylamino)methyliumyl]-3H-benzotriazol-1-oxide hexafluorophosphate
MTBE	methyl *tert*-butyl ether
NMM	*N*-methylmorpholine
NMP	1-Methylpyrrolidin-2-one
TES	triethylsilane
TFA	2,2,2-trifluoroacetic acid
TIPS	triisopropylsilane

## References

[1] Smith W.L., DeWitt D.L., Allen M.L. (1983). Bimodal distribution of the prostaglandin I2 synthase antigen in smooth muscle cells. J. Biol. Chem..

[2] Hara S., Morishita R., Tone Y., Yokoyama C., Inoue H., Kaneda Y., Ogihara T., Tanabe T. (1995). Overexpression of prostacyclin synthase inhibits growth of vascular smooth muscle cells. Biochem. Biophys. Res. Commun..

[3] Todaka T., Yokoyama C., Yanamoto H., Hashimoto N., Nagata I., Tsukahara T., Hara S., Hatae T., Morishita R., Aoki M. (1999). Gene Transfer of Human Prostacyclin Synthase Prevents Neointimal Formation After Carotid Balloon Injury in Rats. Stroke.

[4] Yokoyama C., Yabuki T., Shimonishi M., Wada M., Hatae T., Ohkawara S., Takeda J., Kinoshita T., Okabe M., Tanabe T. (2002). Prostacyclin-deficient mice develop ischemic renal disorders, including nephrosclerosis and renal infarction. Circulation.

[5] Clapp L.H., Gurung R. (2015). The mechanistic basis of prostacyclin and its stable analogues in pulmonary arterial hypertension: Role of membrane versus nuclear receptors. Prostaglandins Other Lipid Mediat..

[6] Cathcart M.-C., Reynolds J.V., O’Byrne K.J., Pidgeon G.P. (2010). The role of prostacyclin synthase and thromboxane synthase signaling in the development and progression of cancer. Biochim. Biophys. Acta.

[7] Sasaki Y., Ochiai T., Takamura M., Kondo Y., Yokoyama C., Hara S. (2017). Role of prostacyclin synthase in carcinogenesis. Prostaglandins Other Lipid Mediat..

[8] Moore P.K. (1982). Prostaglandins, prostacyclin and thromboxanes. Biochem. Educ..

[9] Ueno N., Murakami M., Tanioka T., Fujimori K., Tanabe T., Urade Y., Kudo I. (2001). Coupling between cyclooxygenase, terminal prostanoid synthase, and phospholipase A2. J. Biol. Chem..

[10] Hamberg M., Samuelsson B. (1971). On the Metabolism of Prostaglandins E1 and E2 in Man. J. Biol. Chem..

[11] Cawello W., Schweer H., Müller R., Bonn R., Seyberth H.W. (1994). Metabolism and pharmacokinetics of prostaglandin E1 administered by intravenous infusion in human subjects. Eur. J. Clin. Pharmacol..

[12] Ivanov A.S., Ershov P.V., Molnar A.A., Mezentsev Y.V., Kaluzhskiy L.A., Yablokov E.O., Florinskaya A.V., Gnedenko O.V., Medvedev A.E., Kozin S.A. (2016). Direct molecular fishing in molecular partners investigation in protein–protein and protein–peptide interactions. Russ. J. Bioorg. Chem..

[13] Ivanov A.S., Ershov P.V., Mezentsev Y.V., Poverennaya E.V., Lisitsa A.V., Archakov A.I. (2012). Protocols of protein interactomics: Molecular fishing on optical chips and magnetic nanoparticles. Biochem. Mosc. Suppl. Ser. B.

[14] Ershov P.V., Mezentsev Y.V., Yablokov E.O., Kaluzhskiy L.A., Florinskaya A.V., Gnedenko O.V., Zgoda V.G., Vakhrushev I.V., Raeva O.S., Yarygin K.N. (2018). Direct Molecular Fishing of Protein Partners for Proteins Encoded by Genes of Human Chromosome 18 in HepG2 Cell Lysate. Russ. J. Bioorg. Chem..

[15] Ivanov A.S., Medvedev A., Ershov P., Molnar A., Mezentsev Y., Yablokov E., Kaluzhsky L., Gnedenko O., Buneeva O., Haidukevich I. (2014). Protein interactomics based on direct molecular fishing on paramagnetic particles: Practical realization and further SPR validation. Proteomics.

[16] Svirid A.V., Ershov P.V., Yablokov E.O., Kaluzhskiy L.A., Mezentsev Y.V., Florinskaya A.V., Sushko T.A., Strushkevich N.V., Gilep A.A., Usanov S.A. (2017). Direct Molecular Fishing of New Protein Partners for Human Thromboxane Synthase. Acta Nat..

[17] Ershov P., Mezentsev Y., Gnedenko O., Mukha D., Yantsevich A., Britikov V., Kaluzhskiy L., Yablokov E., Molnar A., Ivanov A. (2012). Protein interactomics based on direct molecular fishing on paramagnetic particles: Experimental simulation and SPR validation. Proteomics.

[18] Wiśniewski J.R., Zougman A., Nagaraj N., Mann M. (2009). Universal sample preparation method for proteome analysis. Nat. Methods.

[19] Pierce B.G., Wiehe K., Hwang H., Kim B.-H., Vreven T., Weng Z. (2014). ZDOCK server: Interactive docking prediction of protein-protein complexes and symmetric multimers. Bioinformatics.

[20] Kim D.E., Chivian D., Baker D. (2004). Protein structure prediction and analysis using the Robetta server. Nucleic Acids Res..

[21] Pettersen E.F., Goddard T.D., Huang C.C., Couch G.S., Greenblatt D.M., Meng E.C., Ferrin T.E. (2004). UCSF Chimera—A visualization system for exploratory research and analysis. J. Comput. Chem..

[22] Florinskaya A.V., Ershov P.V., Mezentsev Y.V., Kaluzhskiy L.A., Yablokov E.O., Buneeva O.A., Zgoda V.G., Medvedev A.E., Ivanov A.S. (2018). The Analysis of Participation of Individual Proteins in the Protein Interactome Formation. Biochem. (Mosc.) Suppl. Ser. B Biomed. Chem..

[23] Florinskaya A., Ershov P., Mezentsev Y., Kaluzhskiy L., Yablokov E., Medvedev A., Ivanov A. (2018). SPR biosensors in direct molecular fishing: Implications for protein interactomics. Sensors (Switzerland).

[24] Ershov P.V., Mezentsev Y.V., Yablokov E.O., Kaluzhskiy L.A., Vakhrushev I.V., Gnedenko O.V., Florinskaya A.V., Gilep A.A., Usanov S.A., Yarygin K.N. (2019). A Method of Lysate Preparation to Improve the Isolation Efficiency of Protein Partners for Target Proteins Encoded by the Genes of Human Chromosome 18. Biomed. Chem. Res. Methods.

[25] Ershov P.V., Mezentsev Y.V., Yablokov E.O., Kaluzhsky L.A., Florinskaya A.V., Buneeva O.A., Medvedev A.E., Ivanov A.S. (2018). Effect of Bioregulator Isatin on Protein–Protein Interactions Involving Isatin-Binding Proteins. Russ. J. Bioorg. Chem..

[26] Barski O.A., Tipparaju S.M., Bhatnagar A. (2008). The Aldo-Keto Reductase Superfamily and its Role in Drug Metabolism and Detoxification. Drug Metab. Rev..

[27] Jakobsson P.J., Morgenstern R., Mancini J., Ford-Hutchinson A., Persson B. (1999). Common structural features of MAPEG—A widespread superfamily of membrane associated proteins with highly divergent functions in eicosanoid and glutathione metabolism. Protein Sci..

[28] Jakobsson P.-J., Thorén S., Morgenstern R., Samuelsson B. (1999). Identification of human prostaglandin E synthase: A microsomal, glutathione-dependent, inducible enzyme, constituting a potential novel drug target. Proc. Natl. Acad. Sci. USA.

[29] Buckley B.J., Kent R.S., Whorton A.R. (1991). Regulation of endothelial cell prostaglandin synthesis by glutathione. J. Biol. Chem..

[30] Walsh S.W., Wang Y. (1993). Deficient glutathione peroxidase activity in preeclampsia is associated with increased placental production of thromboxane and lipid peroxides. Am. J. Obstet. Gynecol..

[31] Bogaards J.J., Venekamp J.C., van Bladeren P.J. (1997). Stereoselective conjugation of prostaglandin A2 and prostaglandin J2 with glutathione, catalyzed by the human glutathione S-transferases A1-1, A2-2, M1a-1a, and P1-1. Chem. Res. Toxicol..

[32] Ellgaard L., Ruddock L.W. (2005). The human protein disulphide isomerase family: Substrate interactions and functional properties. EMBO Rep..

[33] Li H., Yang K., Wang W., Niu Y., Li J., Dong Y., Liu Y., Wang C.-C., Wang L., Liang H. (2018). Crystal and solution structures of human protein-disulfide isomerase-like protein of the testis (PDILT) provide insight into its chaperone activity. J. Biol. Chem..

[34] Yamamoto Y., Takase K., Kishino J., Fujita M., Okamura N., Sakaeda T., Fujimoto M., Yagami T. (2011). Proteomic Identification of Protein Targets for 15-Deoxy-Δ12,14-Prostaglandin J2 in Neuronal Plasma Membrane. PLoS ONE.

[35] Aguiar M., Masse R., Gibbs B.F. (2005). Regulation of Cytochrome P450 by Posttranslational Modification. Drug Metab. Rev..

[36] Madeira F., Tinti M., Murugesan G., Berrett E., Stafford M., Toth R., Cole C., MacKintosh C., Barton G.J. (2015). 14-3-3-Pred: Improved methods to predict 14-3-3-binding phosphopeptides. Bioinformatics.

[37] Zhou S.-N., Lu J.-X., Wang X.-Q., Shan M.-R., Miao Z., Pan G.-P., Jian X., Li P., Ping S., Pang X.-Y. (2019). S-Nitrosylation of Prostacyclin Synthase Instigates Nitrate Cross-Tolerance In Vivo. Clin. Pharmacol. Ther..

[38] Dutka T.L., Mollica J.P., Lamboley C.R., Weerakkody V.C., Greening D.W., Posterino G.S., Murphy R.M., Lamb G.D. (2017). S-nitrosylation and S-glutathionylation of Cys134 on troponin I have opposing competitive actions on Ca2+ sensitivity in rat fast-twitch muscle fibers. Am. J. Physiol. Cell Physiol..

[39] Dietz K.-J. (2003). Redox control, redox signaling, and redox homeostasis in plant cells. Int. Rev. Cytol..

[40] Haunhorst P., Hanschmann E.-M., Bräutigam L., Stehling O., Hoffmann B., Mühlenhoff U., Lill R., Berndt C., Lillig C.H. (2013). Crucial function of vertebrate glutaredoxin 3 (PICOT) in iron homeostasis and hemoglobin maturation. Mol. Biol. Cell.

[41] Li H., Mapolelo D.T., Randeniya S., Johnson M.K., Outten C.E. (2012). Human Glutaredoxin 3 Forms [2Fe-2S]-Bridged Complexes with Human BolA2. Biochemistry.

[42] Cooper A.J.L., Pinto J.T., Callery P.S. (2011). Reversible and irreversible protein glutathionylation: Biological and clinical aspects. Expert Opin. Drug Metab. Toxicol..

[43] Meunier L., Usherwood Y.-K., Chung K.T., Hendershot L.M. (2002). A subset of chaperones and folding enzymes form multiprotein complexes in endoplasmic reticulum to bind nascent proteins. Mol. Biol. Cell.

[44] Askari A., Thomson S.J., Edin M.L., Zeldin D.C., Bishop-Bailey D. (2013). Roles of the epoxygenase CYP2J2 in the endothelium. Prostaglandins Other Lipid Mediat..

[45] Wong P.Y. (1990). Transformation of prostacyclin (PGI2) to a biologically active metabolite: 5(6)-oxido-PGI1 by cytochrome P450-dependent epoxygenase. Adv. Exp. Med. Biol..

[46] Huang Y., Misquitta S., Blond S.Y., Adams E., Colman R.F. (2008). Catalytically Active Monomer of Glutathione S-Transferase π and Key Residues Involved in the Electrostatic Interaction between Subunits. J. Biol. Chem..

[47] Vargo M.A., Nguyen L., Colman R.F. (2004). Subunit interface residues of glutathione S-transferase A1-1 that are important in the monomer-dimer equilibrium. Biochemistry.

[48] Fabrini R., De Luca A., Stella L., Mei G., Orioni B., Ciccone S., Federici G., Lo Bello M., Ricci G. (2009). Monomer-dimer equilibrium in glutathione transferases: A critical re-examination. Biochemistry.

[49] Hearne J.L., Colman R.F. (2006). Catalytically active monomer of class mu glutathione transferase from rat. Biochemistry.

[50] Adler V., Yin Z., Fuchs S.Y., Benezra M., Rosario L., Tew K.D., Pincus M.R., Sardana M., Henderson C.J., Wolf C.R. (1999). Regulation of JNK signaling by GSTp. EMBO J..

[51] Kura T., Takahashi Y., Takayama T., Ban N., Saito T., Kuga T., Niitsu Y. (1996). Glutathione S-transferase-pi is secreted as a monomer into human plasma by platelets and tumor cells. Biochim. Biophys. Acta.

[52] Krissinel E., Henrick K. (2007). Inference of macromolecular assemblies from crystalline state. J. Mol. Biol..

[53] Medvedev A., Buneeva O., Gnedenko O., Ershov P., Ivanov A. (2018). Isatin, an endogenous nonpeptide biofactor: A review of its molecular targets, mechanisms of actions, and their biomedical implications. BioFactors.

[54] Ershov P.V., Mezentsev Y.V., Yablokov E.O., Kaluzhskiy L.A., Florinskaya A.V., Svirid A.V., Gilep A.A., Usanov S.A., Medvedev A.E., Ivanov A.S. (2018). Specificity of Isatin Interaction with Cytochromes P450. Biochem. (Mosc.) Suppl. Ser. B Biomed. Chem..

