# Supplementary material for: Affinity Isolation and Mass Spectrometry Identification of Prostacyclin Synthase (PTGIS) Subinteractome
Source: Biology (Basel). 2019 Jun 20;8(2):49. doi: 10.3390/biology8020049 (PMC6628129; doi:10.3390/biology8020049)
Supplement: Supplementary file 1 [file biology-08-00049-s001.zip › biology-502151supplementary final/Supplementary File 1.doc]

Affinity Isolation and Mass Spectrometry Identification of Prostacyclin Synthase (PTGIS) Subinteractome

Pavel V. Ershov, Yuri V. Mezentsev, Arthur T. Kopylov, Evgeniy O. Yablokov, Andrey V. Svirid, Aliaksandr Lushchyk, Leonid A. Kaluzhskiy, Andrei A. Gilep, Sergey A. Usanov, Alexey E. Medvedev and Alexis S. Ivanov

**Table 1.** Sample preparation of lysate for model SPR experiments.

| **The composition of the analyzed lysate sample (total volume 400 µL)** | |
| --- | --- |
| **Without addition of exogenous PTGIS** | |
| Сontrol | 10 µL lysate (4 mg/mL) + 190 µL 1Х Buffer A (incubation on ice for 1 min) + 200 µL 2Х Buffer B (with 5 μL protease inhibitor cocktail). |
| Acid treated | 10 µL lysate (4 mg/mL) + 90 µL 2Х Buffer A + 100 µL 100 mM HСl (incubation on ice for 1 min) + 30 µL (~ 300 mM) NaOH solution + 170 µL 2X Buffer B (with 5 μL protease inhibitor cocktail). |
| With addition of exogenous PTGIS | |
| Сontrol | 10 µL lysate (4 mg/mL) + 180 µL 1Х Buffer A (incubation on ice for 1 min) + 10 µL PTGIS + 200 µL 2Х Buffer B (with 5 μLprotease inhibitor cocktail) |
| Acid treated | 10 µL lysate (4 mg/mL) + 90 µL 2Х Buffer A + 100 µL 100 mM HСl (incubation on ice for 1 min) + 30 µL (~ 300 mM) NaOH solution + 160 µL 2X Buffer B (with 5 μLprotease inhibitor cocktail) + 10 µL PTGIS |

**Note:** Buffer A - HBS-N buffer (150 mM NaCl, 10 mM HEPES (4-(2-hydroxyethyl)-1-piperazineethanesulfonic acid, pH 7.4); Buffer B - HBS-EP+ buffer (150 mM NaCl, 10 mM HEPES, 2 mM EDTA, 0.05% Tween, pH 7.4) containing 2 mM DTT.

**Table 2.** Sample preparation of lysate for affinity isolation of protein partners of prostacyclin synthase.

| The composition of the analyzed lysate sample (**total** volume 4000 µL) | |
| --- | --- |
| Сontrol | 100 µL lysate (4 mg/mL) + 1900 µL 1Х Buffer A (incubation on ice for 1 min) + 2000 µL 2Х Buffer B (with 20 μL protease inhibitor cocktail). |
| Acid treated | 100 µL lysate (4 mg/mL) + 900 µL 2Х Buffer A + 1000 µL 100 mM HСl (incubation on ice for 1 min) + 300 µL (~ 300 mM) NaOH solution + 1700 µL 2X Buffer B (with 20 μL protease inhibitor cocktail). |

**Note:** Buffer A - HBS-N buffer (150 mM NaCl, 10 mM HEPES (4-(2-hydroxyethyl)-1-piperazineethanesulfonic acid, pH 7.4); Buffer B - HBS-EP+ buffer (150 mM NaCl, 10 mM HEPES, 2 mM EDTA, 0.05% Tween, pH 7.4) containing 2 mM DTT.

**Table 3.** Amino acid residues participating in GSTA1 – GSTA1 interactions predicted by jsPISA 2.0.5 (http://www.ccp4.ac.uk/pisa/) [Krissinel 2007] using cristal structure PDB ID 1k3y.

| I / S | Residue name,  sequence number | H / S | ASA | BSA | ∆G |
| --- | --- | --- | --- | --- | --- |
| I | GLN 67 | H | 92.66 | 68.43 | -0.20 |
| S | THR 68 |  | 30.57 | 0.00 | 0.00 |
| I | ARG 69 | S | 127.98 | 65.52 | -0.17 |
| I | ALA 70 |  | 39.31 | 39.19 | 0.49 |
| S | ILE 71 |  | 1.01 | 0.00 | 0.00 |
| S | LEU 72 |  | 13.06 | 0.00 | 0.00 |
| I | ASN 73 | H | 58.36 | 40.01 | -0.24 |
| I | TYR 74 |  | 73.71 | 64.98 | 0.68 |
| S | ILE 75 |  | 0.17 | 0.00 | 0.00 |
|  | ALA 76 |  | 0.00 | 0.00 | 0.00 |
| I | SER 77 |  | 63.78 | 36.04 | 0.27 |
| I | LYS 78 |  | 82.78 | 10.51 | 0.16 |
| S | TYR 79 |  | 59.73 | 0.00 | 0.00 |
| S | ASN 80 |  | 106.49 | 0.00 | 0.00 |
| S | LEU 81 |  | 12.39 | 0.00 | 0.00 |
| I | TYR 82 |  | 21.27 | 4.17 | -0.05 |
| S | GLY 83 |  | 34.61 | 0.00 | 0.00 |
| S | LYS 84 |  | 162.00 | 0.00 | 0.00 |
| S | ASP 85 |  | 52.89 | 0.00 | 0.00 |

Note: I – interface residue, S – surface residue; H – hydrogen bond, S – salt bridge; ASA – accessible surface area (A^2^); BSA – buried surface area (A^2^); ∆G – hydrophobic effect (kcal/mol).

Partial SPR validation of interactions between prostacyclin synthase and potential protein partners isolated from rat testis tissue lysate.

SPR analysis of the binding of prostacyclin synthase with full-length recombinant proteins as its potential protein partners.

**Figure 1.** SPR sensorgrams of interaction between PTGIS immobilized on the optical chip CM5 with different concentrations of cytochrome P450 2J2 (CYP2J2): (1) - 75 nM, (2) - 150 nM, (3) - 300 nM. PTGIS was covalently immobilized via amino groups of a protein up to 4000 RU (or 4 ng of a protein per 1 mm^2^ of chip surface). 10 mM maleate buffer (pH 6.0) was used as an immobilization buffer. HBS-EP + buffer (10 mM HEPES (pH 7.5), 150 mM NaCl, 1 mM EDTA, 0.05% Tween 20) was used as a running buffer. CYP2J2 solutions were injected for 5 min at a flow rate of 10 μL/min. Association (kon) and dissociation (koff) rate constants of molecular complex were calculated by separate fitting using 1: 1 Langmuir model of surface binding in the Biaevaluation software and the Kd value was calculated by the ratio koff/kon: Kd = 2.0•10^-7^ M, kon = 1.1•10^4^ M^-1^s^-1^, koff = 2.2•10^-3^ s^-1^. Regeneration of the chip surface after each injection of the CYP2J2 was performed with a solution containing 2M NaCl and 0.4% CHAPS for 30 s at a flow rate of 25 μL/min. Biosensor signal presented as a difference between the working channel and control one (without protein).

**Figure 2.** SPR sensorgrams of interaction between the monomeric form of glutathione-S-transferase (GST) immobilized on the optical chip CM5 with different concentrations of prostacyclin synthase (PTGIS): (1) — 0.25 µМ, (2) — 1 µМ, (3) — 2 µМ, (4) — 5 µМ. The dimeric form of GST preparation (Cat. No. G6511, Sigma Aldrich) was initially covalently immobilized on the chip CM5 via amino groups of a protein (about 6300 RU or 6,3 ng of a protein per 1 mm^2^ of chip surface) and the baseline drift did not exceed 1 RU/min. PTGIS injected as analyte (up to 25 µM) did not interact with the dimeric form of GST. Further, the dissociation of the dimeric form of GST to the monomeric form was carried out with a short injection of a solution of 6.25 mM NaOH and 0.5% Tween for 30 s at a flow rate of 25 μL/min. Biosensor signal decreased twice (3200 RU or 3.2 ng protein / mm2 chip surface). HBS-EP + buffer (10 mM HEPES (pH 7.5), 150 mM NaCl, 1 mM EDTA, 0.05% Tween 20) was used as a running buffer. Regeneration of the chip surface after each injection of the PTGIS was performed with a solution containing 10 mM Gly-HCl (pH 3.0) for 20 s at a flow rate of 25 µL/min. Kd value of molecular complex (4.0•10^-7^ M) was calculated by global fitting using 1: 1 Langmuir model of surface binding in the Biaevaluation software.

SPR analysis of the binding of prostacyclin synthase with peptides to its potential protein partners.

**Figure 3.** SPR sensorgrams of interaction between prostacyclin synthase (PTGIS) immobilized on the optical chip CM3 with different concentrations of a peptide from GSTA1 protein (peptide GSTA1_19): (1) — 5 µМ, (2) — 20 µМ, (3) — 40 µМ. PTGIS was covalently immobilized via amino groups of a protein up to 4000 RU (or 4 ng of a protein per 1 mm^2^ of chip surface). GSTA1_19 peptide stock solution was prepared in 100% DMSO. Peptide solutions were injected for 7 min at a flow rate of 10 µL/min. HBS-N buffer (10 mM HEPES (pH 7.5), 150 mM NaCl), containing the same concentration DMSO as in analyte sample, was used as a running buffer. Regeneration of the chip surface after each peptide injection was performed with a solution containing 2M NaCl and 0.4% CHAPS for 30 s at a flow rate of 25 μL/min. Association (kon) and dissociation (koff) rate constants of the molecular complex were calculated by separate fitting using 1: 1 Langmuir model of surface binding in the Biaevaluation software. Apparent Kd values of molecular complex ranged from 1•10^-6^ – 1•10^-5^M were calculated by the ratio koff/kon.

**Figure 4.** SPR sensorgrams of interaction between prostacyclin synthase (PTGIS) immobilized on the optical chip CM3 with different concentrations of a peptide to from GLRX3 protein (peptide GLRX3_18): 20 – 80 µМ. PTGIS was covalently immobilized via amino groups of a protein up to 4000 RU (or 4 ng of a protein per 1 mm^2^ of chip surface). GLRX3_18 peptide stock solution was prepared in HBS-N buffer. Peptide solutions were injected for 7 min at a flow rate of 10 µL/min. HBS-N buffer (10 mM HEPES (pH 7.5), 150 mM NaCl) was used as a running buffer. Regeneration of the chip surface after each peptide injection was performed with a solution containing 2M NaCl and 0.4% CHAPS for 30 s at a flow rate of 25 μL/min. Association (kon) and dissociation (koff) rate constants, as well as Kd value, were calculated by global fitting using 1: 1 Langmuir model with drifting baseline in the Biaevaluation software. Apparent Kd value was about 19 µM.

**Figure 5.** SPR sensorgrams of interaction between prostacyclin synthase (PTGIS) immobilized on the optical chip CM3 with different concentrations of a peptide from AKR1A1 protein (peptide AKR1A1_18): 2.5 – 20 µМ. PTGIS was covalently immobilized via amino groups of a protein up to 4000 RU (or 4 ng of a protein per 1 mm^2^ of chip surface). AKR1A1_18 peptide stock solution was prepared in HBS-N buffer. Peptide solutions were injected for 7 min at a flow rate of 10 µL/min. HBS-N buffer (10 mM HEPES (pH 7.5), 150 mM NaCl) was used as a running buffer. Regeneration of the chip surface after each peptide injection was performed with a solution containing 2M NaCl and 0.4% CHAPS for 30 s at a flow rate of 25 μL/min. Association (kon) and dissociation (koff) rate constants? as well as Kd value were calculated by global fitting using 1: 1 Langmuir model of surface binding in the Biaevaluation software. Apparent Kd value was about 49 µM.

**Figure 6.** SPR sensorgrams of interaction between prostacyclin synthase (PTGIS) immobilized on the optical chip CM3 with different concentrations of a peptide from AKR1A1 protein (peptide AKR1A1_18(2)): 2.5 – 22.5 µМ. PTGIS was covalently immobilized via amino groups of a protein up to 4000 RU (or 4 ng of a protein per 1 mm^2^ of chip surface). AKR1A1_18(2) peptide stock solution was prepared in HBS-N buffer. Peptide solutions were injected for 7 min at a flow rate of 10 µL/min. HBS-N buffer (10 mM HEPES (pH 7.5), 150 mM NaCl) was used as a running buffer. Regeneration of the chip surface after each peptide injection was performed with a solution containing 2M NaCl and 0.4% CHAPS for 30 s at a flow rate of 25 μL/min. Association (kon) and dissociation (koff) rate constants, as well as Kd value, were calculated by global fitting using 1: 1 Langmuir model of surface binding in the Biaevaluation software. Apparent Kd value was about 47 µM.
